# Supplementary material for: On-surface synthesis and spontaneous segregation of conjugated tetraphenylethylene macrocycles
Source: Commun Chem. 2022 Dec 22;5:174. doi: 10.1038/s42004-022-00794-1 (PMC9814618; doi:10.1038/s42004-022-00794-1)
Supplement: Supplementary file 2 — supplementary materials [file 42004_2022_794_MOESM2_ESM.pdf]

Supplementary information for

On-surface synthesis and spontaneous segregation of conjugated tetraphenylethylene macrocycles

En Li[1], Cheng-Kun Lyu[1], Chengyi Chen[1], Huilin Xie[2], Jianyu Zhang[2], Jacky Wing Yip Lam[2], Ben Zhong Tang[2,3], Nian Lin[1]*

[1]Department of Physics, The Hong Kong University of Science and Technology, Clear Water Bay, Hong Kong, China

[2]Department of Chemistry and the Hong Kong Branch of Chinese National Engineering Research Center for Tissue Restoration and Reconstruction, The Hong Kong University of Science and Technology, Clear Water Bay, Hong Kong, China

[3]School of Science and Engineering, Shenzhen Institute of Aggregate Science and Technology, The Chinese University of Hong Kong, Shenzhen, Guangdong, China

*Correspondence to: phnlin@ust.hk

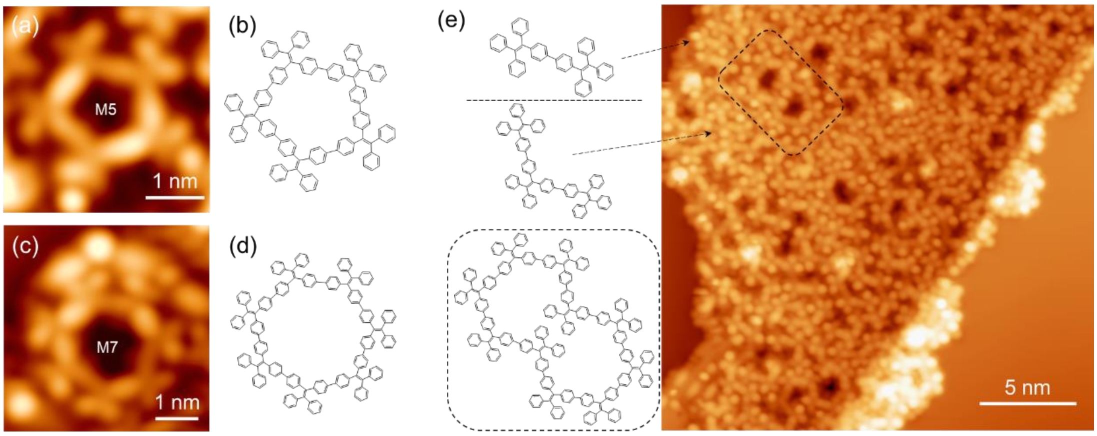

Supplementary Figure 1: (a, b) High-resolution STM image (−0.35V, 100 pA) and chemical model of an M5 macrocycle. (c, d) High-resolution STM image (−0.5V, 100 pA) and chemical model of an M7 macrocycle. (e) Right panel: STM image (−1.15 V, 100 pA) showing disordered mixture of oligo-TPE chains, and odd-number macrocycles. The dashed rectangle marks a Cassini oval-shaped M10 macrocycle. Left panel: corresponding chemical models of dimer, trimer, and M10.

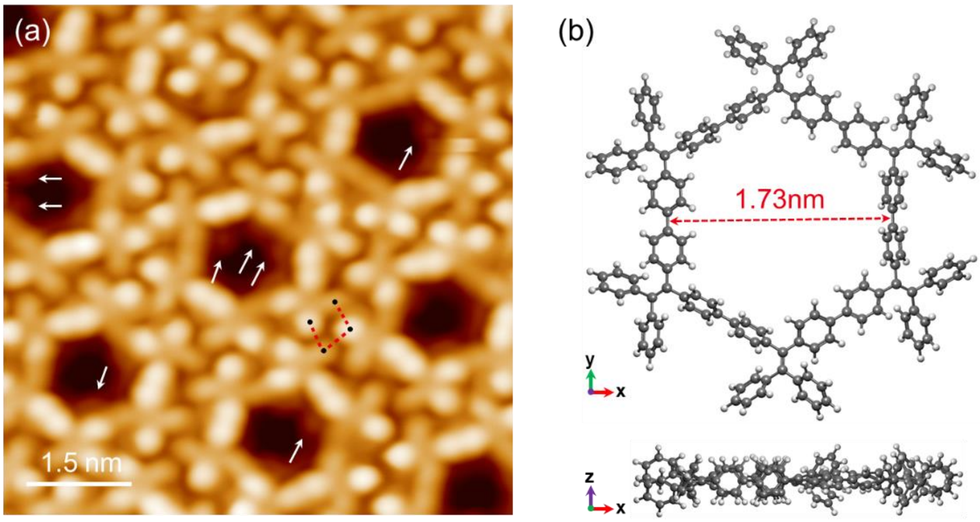

Supplementary Figure 2: (a) High-resolution STM image (−0.55V, 50 pA) of an M6 island. The protrusions inside the cavities, indicated by the white arrows, are attributed to Br adatoms The measured distance between neighboring exterior phenyl groups, indicated by the dashed red lines, is 0.52±0.02 nm. (b) Structure of a planar M6 conformer optimized by DFT method at B3LYP/6-31G(d,p) level, Gaussian 09 program. The side-to-side length of the hexagon is 1.73nm, agreeing well with the measured value (1.69±0.03 nm).

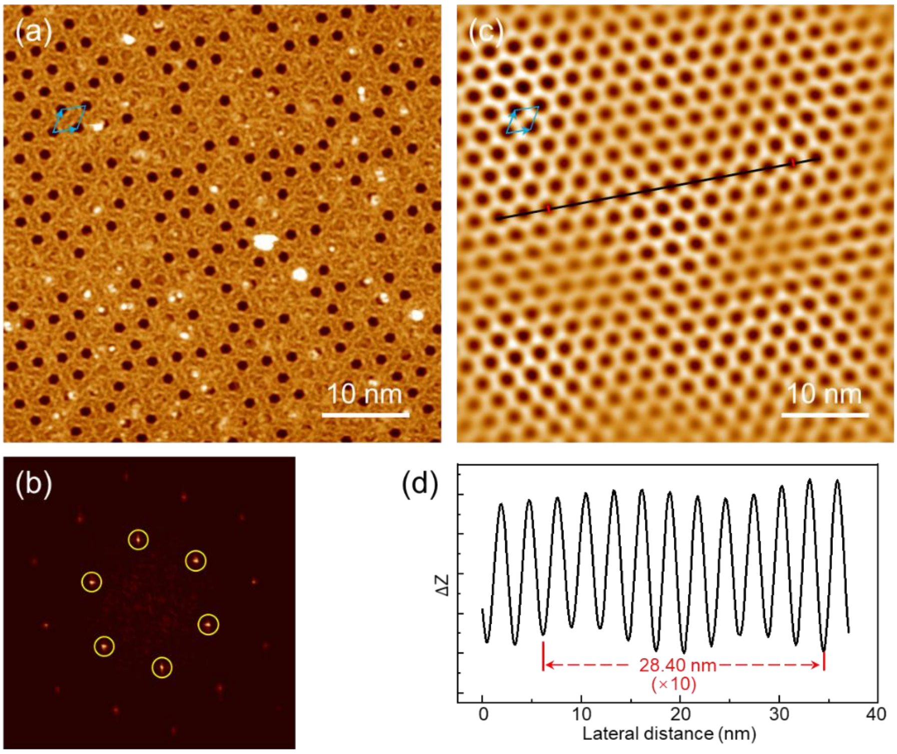

Supplementary Figure 3: (a) STM topography (−1.8V, 10 pA) of an M6 island. (b) Fast Fourier transform (FFT) image of (a). (c) Real space image after filtering and inverse FFT based on the circled Bragg peaks in the FFT shown in (b). The blue rhombic frames in (a) and (c) mark the same position, denoting the unit cell. (d) Line profiles along the black line in (c), showing a measured period of 2.84 nm.

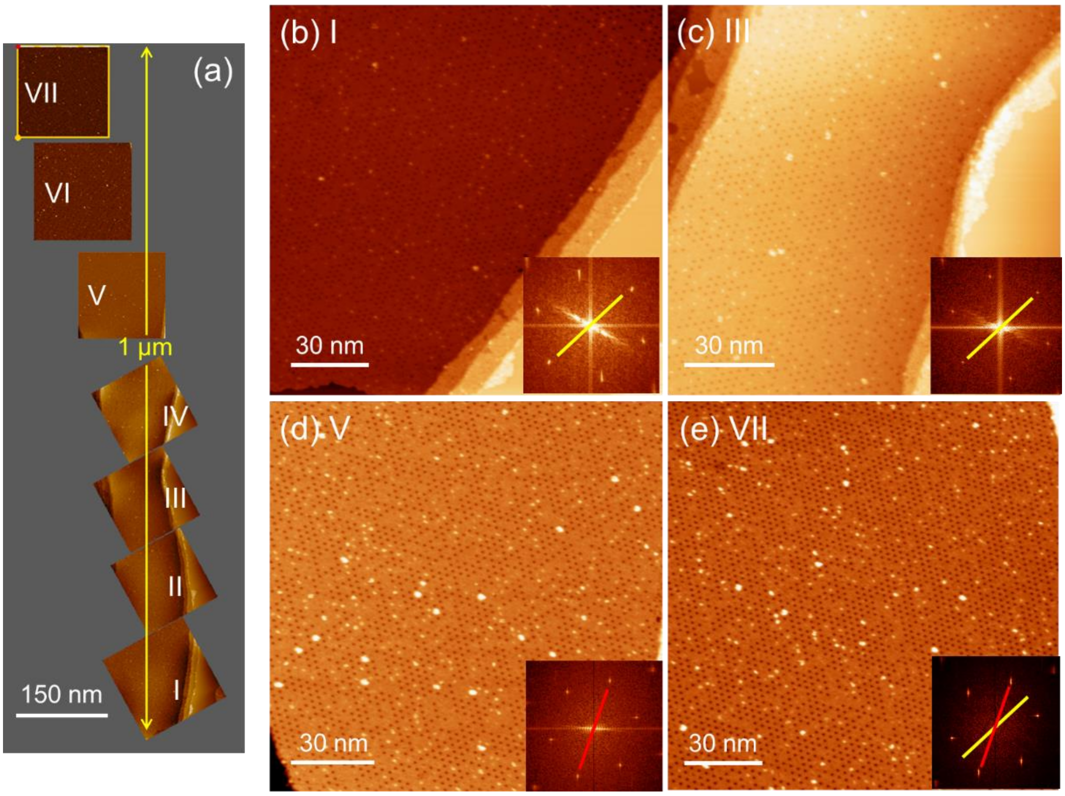

Supplementary Figure 4: (a) A series of large-scale STM images taken along a terrace on the M6 2D crystal, with a total length of more than 1 μm. The relative angle between I-IV and V-VII is 28°. (b-e) Corresponding STM images (−1.0 V, 40 pA) with FFT patterns (inset) labeled in (a). The yellow/red lines mark the orientation of the hexagonal network in region I-III/V-VI, respectively. The measured angle between them is around 28°, indicating that all images are taken on a single domain.

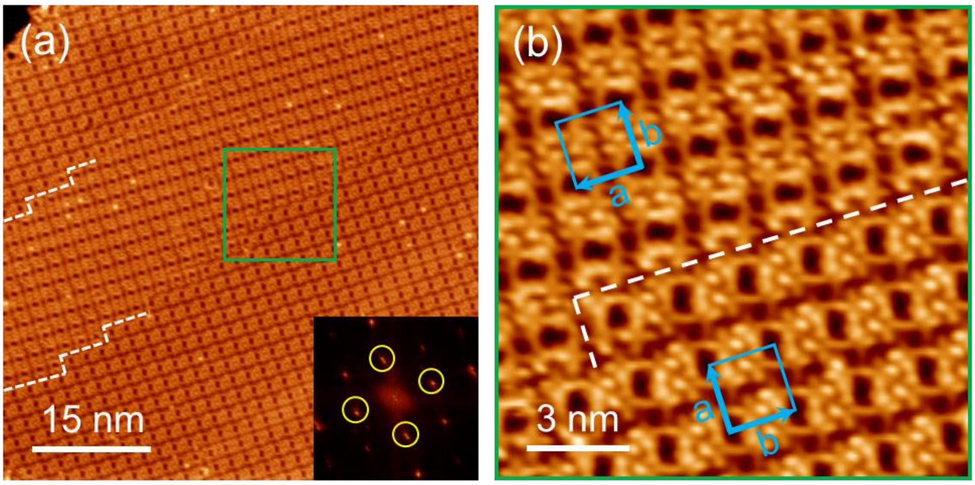

Supplementary Figure 5: (a) Large-scale STM image (V=−0.9 V, I=50 pA) of an M4 island. Inset: FFT pattern. (b) Zoom in STM image of the M4 2D crystal with a domain boundary (dashed line). The blue rectangles denote the unit cell of the 90° rotated domains.

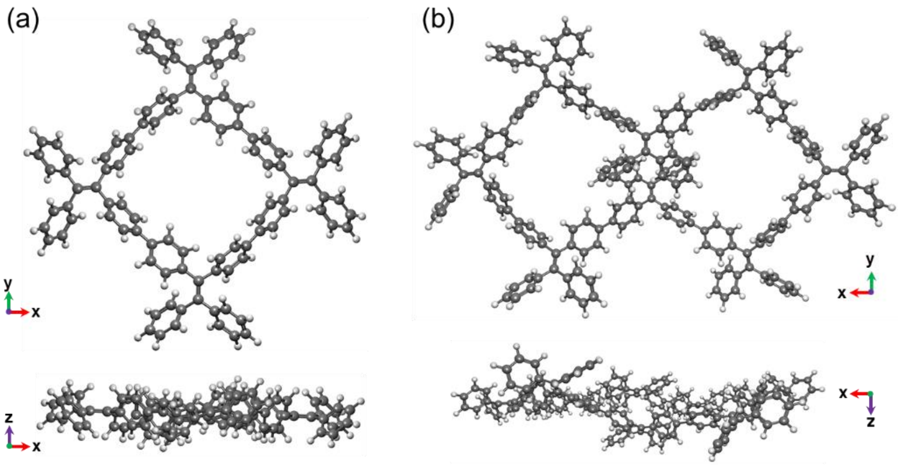

Supplementary Figure 6: (a) Top and side views of a DFT optimized M4 conformer, displaying a planar configuration with tilted exterior phenyl groups. (b) Top and side views of a DFT optimized M8 conformer. Two inward TPE units are spatially separated, resulting in a nonplanar configuration.

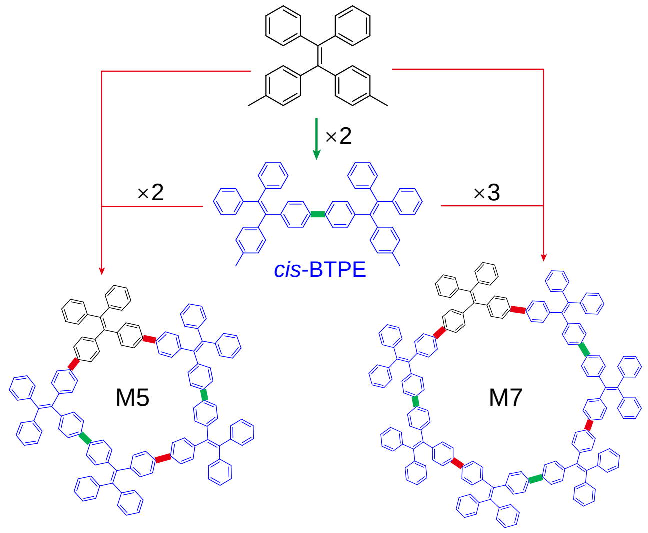

Supplementary Figure 7: Proposed reaction pathways for forming M5 and M7 macrocycles.

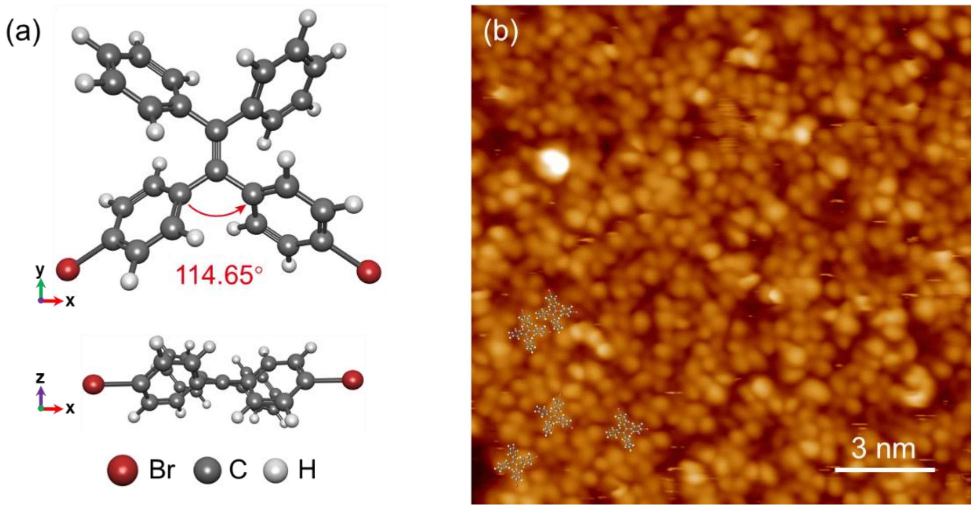

Supplementary Figure 8: (a) DFT optimized geometry of Br_2-TPE, displaying a propeller-like structure. The angle between two phenyl rings connected with the central ethylene, as marked by a red arc arrow, is ~114.65°. (b) STM image (−1.5V, 51 pA) of Br_2-TPE precursors deposited onto Ag(111) held at room temperature, which produces disordered islands. Structural models are superimposed on the image.
